# Biogenic Hydrogen Conversion of De-Oiled Jatropha Waste via Anaerobic Sequencing Batch Reactor Operation: Process Performance, Microbial Insights, and CO_2_ Reduction Efficiency

**DOI:** 10.1155/2014/946503

**Published:** 2014-02-05

**Authors:** Gopalakrishnan Kumar, Chiu-Yue Lin

**Affiliations:** ^1^Department of Environmental Engineering and Science, Feng Chia University, Taichung 40724, Taiwan; ^2^Laboratory for Research on Advanced Processes for Water Treatment, Instituto de Ingeniería, Unidad Académica Juriquilla, Universidad Nacional Autónoma de México, Boulevard Juriquilla 3001, QRO 76230, Mexico; ^3^Green Energy Development Center, Feng Chia University, Taichung 40724, Taiwan; ^4^Master's Program of Green Energy Science and Technology, Feng Chia University, Taichung 40724, Taiwan

## Abstract

We report the semicontinuous, direct (anaerobic sequencing batch reactor operation) hydrogen fermentation of de-oiled jatropha waste (DJW). The effect of hydraulic retention time (HRT) was studied and results show that the stable and peak hydrogen production rate of 1.48 L/L∗d and hydrogen yield of 8.7 mL H_2_/g volatile solid added were attained when the reactor was operated at HRT 2 days (d) with a DJW concentration of 200 g/L, temperature 55°C, and pH 6.5. Reduced HRT enhanced the production performance until 1.75 d. Further reduction has lowered the process efficiency in terms of biogas production and hydrogen gas content. The effluent from hydrogen fermentor was utilized for methane fermentation in batch reactors using pig slurry and cow dung as seed sources. The results revealed that pig slurry was a feasible seed source for methane generation. Peak methane production rate of 0.43 L CH_4_/L∗d and methane yield of 20.5 mL CH_4_/g COD were observed at substrate concentration of 10 g COD/L, temperature 30°C, and pH 7.0. PCR-DGGE analysis revealed that combination of celluloytic and fermentative bacteria were present in the hydrogen producing ASBR.

## 1. Introduction

Two crucial factors that play important role towards sustainable development for the global prosperity are continuous energy supply and environmental-related issues. Energy derived from fossil fuels is dominating the energy sector in recent decades; however, depletion of these reservoirs has made an urge to find alternative fuel sources to fulfill the world's energy demand which would become a big issue in the near future. Among the proposed alternative fuels, such as hydrogen, ethanol, butanol, and methane, hydrogen stands as an extraordinary and promising fuel mainly due to its unique characters like high energy yield (122 kJ/g) and water vapor release upon combustion which are representing the carbon neutral property. In addition, hydrogen use in fuel cells for the production of electricity has been demonstrated widely around the globe [1, 2].

Lignocellulose or solid wastes are proven to be a promising feedstock for biological hydrogen production by various research groups, because of their vast availability, easy collection process, and high content of cellulose (a feasible substrate for hydrogen producing microorganisms) [3–6]. The biodiesel energy sector generates an enormous amount of solid waste especially when jatropha biomass is used as feedstock. The recalcitrant nature and poisonous substances present in this solid waste make the treatment process not economically feasible [7, 8]. Thus, the utilization of this waste for hydrogen fermentation has dual benefits: waste management and energy generation (mainly hydrogen gas, additionally, ethanol and volatile fatty acids would be also generated as fermentation coproducts).

Development of hydrogen fermentation process for lignocellulose waste needs proper operational conditions and strategies; for example, with such solid nature, performing continuous operation (CSTR) would be of great difficulty due to the possibilities of improper flow in the pump and/or pipelines, in other terms the accumulation of solids in a period of time. Thus, ASBR (anaerobic sequencing batch reactor) operation has been suggested and shown as a promising way to treat such solid biomass feedstock and pure substrate such as starch [9–11]. The generation of DJW is abundant, due to the expansion of biodiesel industries. Besides a solid portion of 3 ton remained in every ton of biodiesel extraction. So, development of a hydrogen fermentation form DJW could attain more interest than other lignocellulose feedstock. The main advantages of this feedstock are the easy collection and desizing processes, whereas other feed stocks, like rice straw, wheat straw, corn, and so on, require energy during the size reduction. Moreover, development of hydrogen fermentation for DJW increases the commercial benefits of the biodiesel industry whereas both biodiesel and biohydrogen could be obtained from a low-cost waste, that is, jatropha, at one time input. Thus, here we demonstrated a process to treat DJW using ASBR operation. In addition, the feasibility of the effluent from H_2_ fermentor to generate methane to enhance the total energy production is also evaluated.

## 2. Materials and Methods

### 2.1. DJW Substrate and Anaerobic Mixed Microflora

DJW used in this study was collected from a biodiesel industry using Jatropha biomass and located in central Taiwan. The cellulosic content was analyzed as 42.3% of fermentable sugars (14.1% cellulose and 28.2% of hemicellulose) using FIBERTEC 1020 (M6) analyzer as mentioned elsewhere [12]. Anaerobic mixed microflora was obtained from a municipal wastewater treatment plant. Substrate and seed sludge were stored in a refrigerator at 4°C before being used in the experiments. To inactivate the hydrogen consuming methanogens, the sludge was heat treated for 30 minutes at 95°C in a boiling water bath. Characteristics of the DJW and seed sludge were described in our previous study [12].

### 2.2. Reactor (ASBR) Startup and Operation

**Figure 1:**
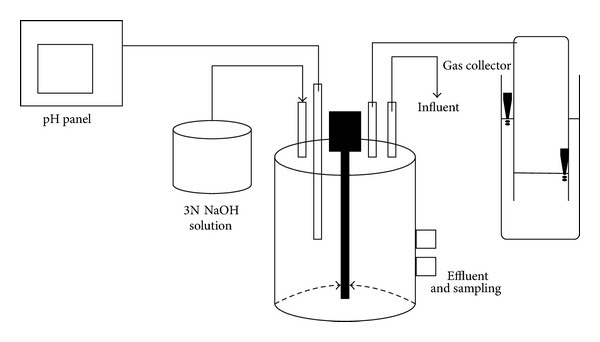
ASBR schematic diagram.

A schematic diagram of the ASBR is shown in Figure 1. The reactor was started up by feeding glucose (10 g/L) initially to enrich the hydrogen producers (Run 1). There forward, DJW at a concentration of 100 g/L was fed for the adaptation of hydrogen producers at an agitation speed of 150 rpm and operational temperature of 55°C. Meanwhile, the effluent was collected and fed into the reactor along with fresh substrate to avoid the loss of biomass in the form of effluent (Run 2–4). After Run 4, it was stopped due to the accumulation of more amounts of solid particles inside the reactor. Thereafter, only fresh substrate was fed into the reactor. The nutrient solution added was prepared by following the Endo formulation [13] with slight modifications as mentioned [11]. ORP and pH were monitored using an automated pH, ORP panel; pH was controlled using 1 N NaOH buffer.

### 2.3. Batch Methane Fermentation

Methane production was studied in batch vials (capacity-125 mL) with a working volume of 60 mL by adding 40 mL of hydrogen fermentation effluent (substrate concentration 10 g COD/L), 12 mL of seed sludge (either cow dung or pig slurry), 1–3 mL for pH adjustment (either 1 N NaOH or HCl), 5 mL nutrient solution (to enhance the growth of anaerobic bacteria), and the rest was tap water. The nutrient solution added was prepared by following the Endo formulation as mentioned [13]. The final pH was adjusted to neutral (7.0). Then, the batch vials were kept in a reciprocal air-bath shaker at 150 rpm at 30°C. The volume and composition of gas were monitored daily. Fermentation was carried out until the gas production becomes zero. All experiments were carried out at least triplicates and the values represented are the mean of triplicate values.

### 2.4. Analytical Procedures

The analytical procedures used to determine pH, total solid (TS), COD, and VSS were followed by APHA 1995 [14]. The volatile fatty acids (VFAs) and ethanol concentrations were analyzed by HPLC. The gas composition was analyzed with a gas chromatograph having a thermal conductivity detector (China Chromatograph 8700T). Other experimental procedures were indicated in our previous studies [15, 16]. For total carbohydrate concentration, anthrone-sulphuric acid method was adapted [17]. Biogas production was measured in a periodic interval using various volumes of glass syringes, depending on the expected biogas production fitted with hypodermic needles as described by Owen et al. [18].

### 2.5. Calculations

The modified Gompertz equation was used to estimate methane production potential and methane production rate (Sigma plot software 10.0, Systat Software Inc., USA):

(1) $$\begin{array}{c} H\left(t\right)=P\cdot \exp\left\{-\exp\left[\frac{R_{m}\cdot e}{P}\left(\lambda -t\right)+1\right]\right\}, \end{array}$$

where *H*(*t*) is the cumulative methane production (mL); *P* is the methane production potential (mL); *R*_*m*_ is the maximum methane production rate (mL/h); *e* is 2.71828; *λ* is the lag phase time (h); and *t* is the cultivation time (h). Methane production rate (MPR, L H_2_/L-d) was defined as the value of *R*_*m*_ divided by the reactor volume (0.06 L) and multiplied by one day (24 h); methane yield (MY, mL CH_4_/g COD) was defined as methane produced per gram chemical oxygen demand (COD).

### 2.6. PCR-DGGE, DNA Sequencing, and Phylogenetic Analysis

Total genomic DNA was isolated using the Blood & Tissue Genomic DNA Extraction, Miniprep System (Viogene, Taiwan), following the manufacturer's instructions. The isolated DNA was confirmed by agarose gel electrophoresis (0.75%) and the samples were stored at −20°C for further PCR reactions. The PCR mixtures (50 *μ*L) contained each deoxynucleoside triphosphate at a concentration of 200 mM, 1.5 mM MgCl_2_, each primer at a concentration of 0.2 mM, 1.25 U of Taq DNA polymerase (Promega, Madison, WI, USA), and the PCR buffer provided with the enzyme. The amplification consisted of a DNA denaturing step at 94°C for 5 min, followed by 30 cycles of denaturation at 94°C for 1 min, 1 min annealing at 55°C for EUB968gc-UNIV1392r, and extension at 72°C for 1 min. The cycling included a final extension step at 72°C for 10 min to ensure full extension of the product. All PCR operations were performed with an automatic thermal cycler iCyclerTM (Bio-Rad, CA, USA). PCR products were analyzed by electrophoresis at 100 V for 30 min through 1.5% (wt/vol) agarose gel. The amplified PCR products were used for denatured gradient gel electrophoresis (DGGE) analysis. The DGGE profile of the PCR-amplified DNA was obtained following the method mentioned [19] using a DCode Universal Mutation Detection System (BioRad, USA). The 6% (w/v) of acrylamide solution was used to cast a gel with denaturant gradients ranging from 40% to 60%. Electrophoresis was conducted in a 1X TAE (Tris/acetic acid/EDTA) buffer solution at 80 V and 60°C for 12 h. The gels were stained for 10 min with ethidium bromide and visualized under UV radiation. The number of operational taxonomic units (OTU) for each sample was defined as the number of DGGE bands. The selected DGGE bands on the gel was excised with a sterile razor blade, placed in 1.5 mL centrifuge tube and add 50 *μ*L of sterile 1X TAE buffer, and then incubated overnight at 4°C to reclaim the DNA. Additional PCR-DGGE analyses were performed to ensure the purity of reclaimed DNA. Analysis of targeted DNA sequences was performed in a DNA sequencer (Tri Biotech, Taiwan). The bioinformatic analysis was carried out by using the tool BLASTN facility available from NCBI website (http://www.ncbi.nlm.nih.gov/BLAST/) to align the partial 16S rDNA sequences with the reference microorganisms available in the GenBank database. These sequences were further aligned with the closest matches found in the GenBank Database with the CLUSTALW function of MEGA 4 [20].

### 2.7. Accession Numbers

16s rDNA (4) sequences were submitted to GenBank and the accession numbers of the gene sequences submitted to GenBank included KC503758-KC503761.

## 3. Results

### 3.1. Hydrogen Production Performance in ASBR: Effect of HRT

**Figure 2:**
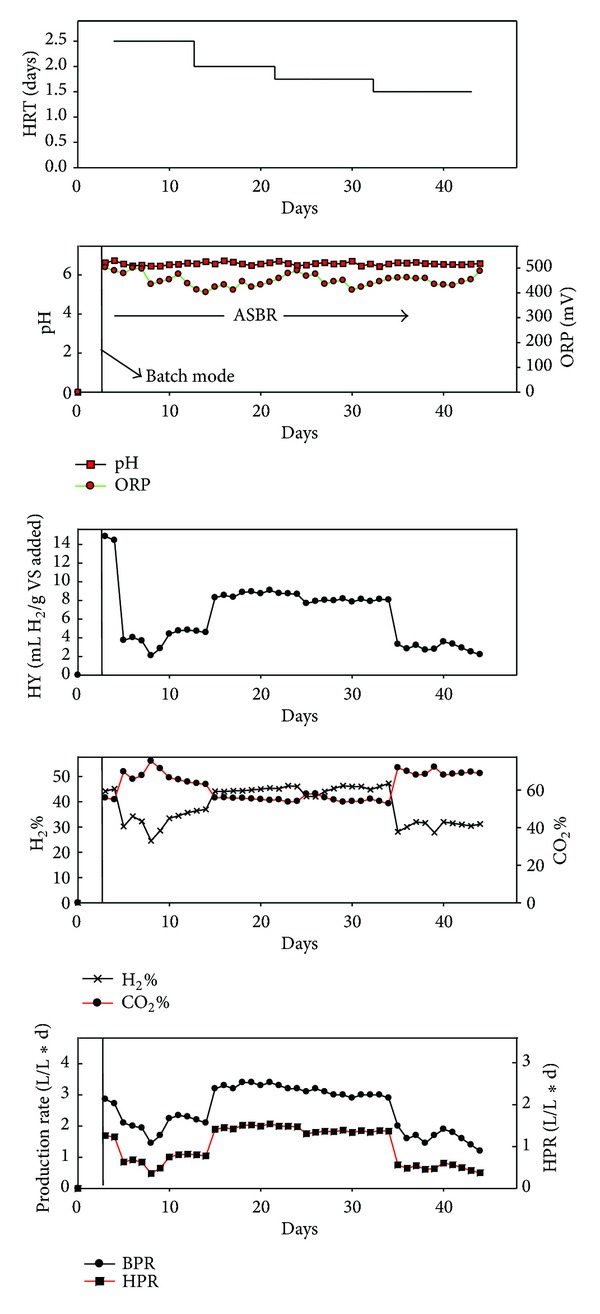
ASBR reactor performances.

The production performance of the ASBR is shown in Figure 2. The performance has shown fluctuations during the start-up period. However, a steady state condition has been reached after 30 days, while the HRT was 2 days. Initially, the reactor was fed with glucose and DJW (100 g/L) in a batch mode to stabilize and enrich the microbial population in order to utilize the complex substrate. The adaptation of microbes to the new environment after 15 days attributed to the gradual increase in hydrogen production. The operation strategies were presented in Table 1. Shortened HRT has enhanced performance of the hydrogen production rate (HPR) from 0.56 ± 0.13 L/L-d to 1.48 ± 0.04 L/L-d.

The biogas production rate (BPR) and hydrogen content were shown in Table 2. ASBR strategy has been previously reported as it could increase the biomass concentration. Usually biomass loss occurs in the CSTR when operated at lower HRT. This is the reason many authors have suggested ASBR operation for better performances, especially while solid substrate is used, in order to avoid the blockings in the pump during the continuous feeding [9–11]. The main advantage of thermophilic temperature in hydrogen production is the reduced solubility of hydrogen which would lower the hydrogen inhibition. Thus, thermophilic temperature opted in this study also contributes towards the better performance. The feed provided intermittently to the reactor had avoided the oxygen inhibition from the influent. This proved the potential of this system and turned as the peak HPR of 1.48 ± 0.04 L/L∗d. However, the yield is still low at steady state conditions, and it is similar to our previous study which provided the same results in batch experiments [12]. Peak hydrogen yield (HY) of 8.7 ± 0.3 mL H_2_/g VS added was observed at steady state conditions.

### 3.2. Soluble Metabolites

The soluble metabolic products (SMP) analysis at the steady state condition revealed that acetic and butyric acids were the main intermediates produced during the DJW fermentation; besides propionate and ethanol were detected at low level. Mainly propionic acid and lactic acid are considered as an undesirable side-product of dark-fermentative biohydrogen technology. Butyric acid was detected at higher amount (2.3 ± 0.4 g/L), revealing that the fermentation was mediated through butyrate which is favorable for hydrogen production as reported in other studies (Table 3). Such an acid dominated pathway led to the efficient bioH_2_ production in many other studies, as well in [15, 16]. The effluent has been collected and it comprised of about 9.3 ± 1.8 g/L of total carbohydrate and 14.4 ± 2.1 g COD/L as total chemical oxygen demand. Total solids was also shown (Table 3). Similar kinds of results were reported during ASBR operation of marine algae. Besides, CH_4_ fermentation of the H_2_ fermentation effluent is suggested in order to increase the total energy production of the process [9].

### 3.3. CH_4_ Fermentation via Hydrogen Fermentation Effluent

**Figure 3:**
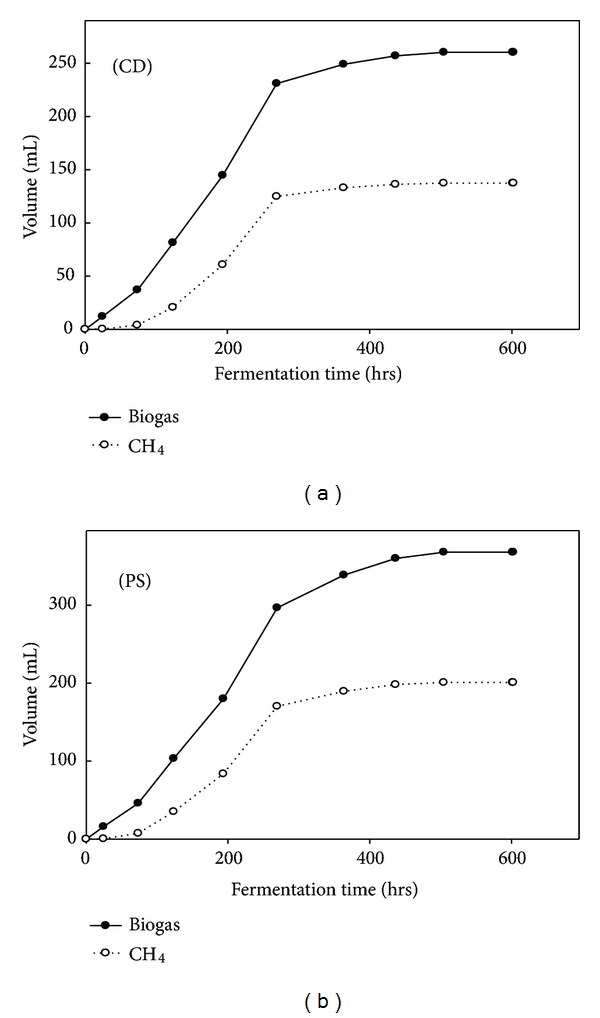
Batch CH_4_ fermentation profiles of pig slurry (PS) and cow dung (CD).

The CH_4_ batch fermentation results showed that the effluent could be digested and converted into methane. Peak MPR and MY of 425.3 ± 5.1 mL CH_4_/L∗d and 20.5 ± 0.5 mL CH_4_/g COD were attained while using pig slurry as a seed source (Table 4). The results are presented in Table 4. Cow dung also provided a MPR and MY of 348.6 ± 4.2 mL CH_4_/L∗d and 13.7 ± 0.8 mL CH_4_/g COD, respectively. The biogas and methane production profile of both the seed sources (cow dung and pig slurry) were shown in Figure 3. Pig slurry has been shown as a good seed source for methane fermentation than cow dung for the effluent from hydrogen producing ASBR using DJW. These results indicate that the process proposed could enhance the energy production of the total process.

### 3.4. Microbial Community Composition

**Figure 4:**
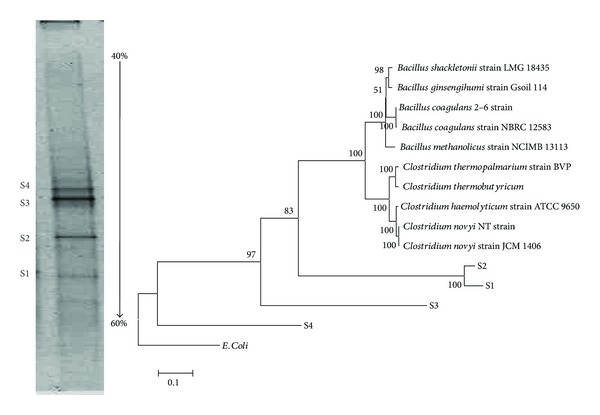
PCR-DGGE profile of the microbial community and Phylogenetic tree of the respective OTUs.

In order to detect the dominant microorganisms present in the reactor during the steady state operation samples were taken at 34th day of operation, while the hydrogen production was shown as the maximal. DGGE band pattern has been obtained by using the primer set EU968gc-UNIV1392r could reveal the structure composition of the microbial communities in the mixed cultures and is based on the V6 region of the 16s rRNA gene. Based on DGGE profile 4 distinct bands were noted. These bands were excised and purified to determine their 16s rRNA sequencing analysis as shown in Table 5 and Figure 4. The evolutionary history was inferred using the Neighbor-Joining method [21]. A total of 4 operational taxonomic units (OTU) were obtained (Table 5) in which 2 of them belonged to the phyla Firmicutes. All the bacteria were distantly related with >95% to *Clostridium sensu stricto *such as *Clostridium thermopalmarium. *The other 2 bands also belonged to the same phyla, but, the species are identified as *Bacillus ginsengihumi* and *Bacillus coagulans*.

## 4. Discussions

### 4.1. Effect of HRT on Process Performance

During ASBR operation, the gaseous components were analyzed as H_2_ and CO_2_ and methane was not detected until the end of fermentation. This indicates that the heat treatment of sewage sludge strongly suppressed the methanogenic activity as we mentioned in our previous study [22]. Reducing hydraulic retention time (HRT) resulted in the enhanced hydrogen production performance [11] and is also one of the methods to develop a particular group of stable hydrogen producers. The production performance shown in this study could be supported by other studies that employed similar ASBR operation for lignocellulose-based waste such as marine algae and sweet sorghum syrup [9, 23]. The yield is relatively low in the fermentative hydrogen production process as discussed earlier [24]. Generally, in dark fermentation the maxima yield that could be achieved is only 33% even pure sugar such as glucose is used, the main reason for this drawback is the distribution of electrons to other intermediate products such as acetate, where only 10% of the stoichiometry could be achieved while the substrate conversion rate is more than 98%. In our study the substrate conversion rate in terms of total carbohydrate is only about 50% as indicated in our previous report [12].

Generally, hydrogen fermentation is associated with the production of intermediate acid production. The production of VFAs or solvents during the anaerobic fermentation process is often a crucial signal in monitoring the feasibility of hydrogen producing cultures [25, 26]. While glucose is used as a substrate the maximum theoretical yields of 4 mol and 2 mol hydrogen would be produced via acetic and butyric acid pathways as shown in (2) and (3), respectively,

(2) $$\begin{array}{c} {\text{C}}_{6}{\text{H}}_{12}{\text{O}}_{6}+2{\text{H}}_{2}\text{O}⟶2{\text{CH}}_{3}\text{COOH}+2{\text{CO}}_{2}+4{\text{H}}_{2}, \end{array}$$

(3) $$\begin{array}{c} {\text{C}}_{6}{\text{H}}_{12}{\text{O}}_{6}⟶{\text{CH}}_{3}{\text{CH}}_{2}{\text{CH}}_{2}\text{COOH}+2{\text{CO}}_{2}+2{\text{H}}_{2} \end{array}$$

In recent years the hydrogen fermentation effluent is utilized for the production of methane or hydrogen by means of anaerobic digestion or photofermentation as it could effectively add more amount of energy to the process [9, 27]. The effluent (rich in organic acids) was utilized for methane fermentation since many studies reveal that VFAs are a good source for methane fermentation especially in two-stage fermentation [28]. Thus, the effluent was employed in batch reactors to generate methane using two types of seed inoculum as cow dung and pig slurry. In fact, cow dung and pig slurry are good source for methane generation as indicated in other studies [28, 29]. In our study also we have demonstrated that hydrogen fermentation effluent has the potential for the methane generation, which in turn increases the total energy efficiency of the process.

### 4.2. Microbial Insights during the ASBR Operation

In the microbial insights responsible for hydrogen fermentation, two bands were closely related to* Clostridium thermopalmarium* reported as the potential hydrogen producing bacterium [30] which produces hydrogen from cellulose, since it contains cellulolytic enzymes. The strains of genus *Clostridium* are able to produce acetate and butyrate as well as hydrogen during anaerobic fermentation using glucose as substrate [31]. Besides, composition of DJW is mainly of cellulose (polymer of glucose) and hemicellulose (such as xylose, arabinose, and cellobiose) [22]. The other genus belongs to Bacillus and the same phyla Firmicutes, and the species were identified as *Bacillus ginsengihumi *and* Bacillus coagulans*. *Bacillus coagulans* is reported as the producer of lactic acid from hemicellulose extracts [32] at slightly thermophilic temperature, due to these reasons only these two major organisms were present in the reactor during the steady state conditions. The inoculum source (sewage sludge) selected in this present study was a rich source of hemicelluloytic and cellulolytic bacteria, as reported previously [33]. The PCR-DGGE based sequence analysis revealed the presence of dominant butyrate mediated hydrogen producing bacteria present in the reactor at steady state. However, the PCR-DGGE does not reveal the quantity of the microbial populations used for the qualitative analysis of the organism's identification. The heat treatment method has been applied mostly for eliminating homoacetogens with consequent microbial community reduction. Though many nonspore hydrogen producers could be destroyed by heat, it enhances the growth of *Clostridial *spp. which in turn results in higher hydrogen production efficiency. Moreover, *Bacillus* is also reported as spore formers while the unfavorable conditions occurred. Thus, the combination of these cellulolytic and fermentative bacteria supported the possible pathway of hydrogen generation.

### 4.3. COD Balance and CO_2_ Reduction Efficiency

**Figure 5:**
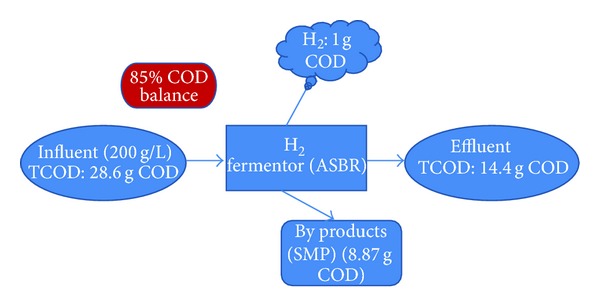
COD balance of the ASBR system.

The COD balance of the system has been shown in the Figure 5. It can be seen that nearly 85% of the COD has been balanced, the remaining percentage would be the trace amount of SMPs (like butanol, vareate, etc.), which were not detected by the GC-FID. Peak HPR and HY of 7.3 L/Kg-d and 8.3 L/Kg DJW were attained during the ASBR operation, which accounts for the energy production of 9.4 Gj/ha/y for the biohydrogen production (carbon neutral). According to our previous study [28], by replacing the hydrogen energy produced from this process, the amount of CO_2_ reduction was analyzed as 0.25 tons for coal, 0.2 tons for fuel oil, and 0.17 tons for the natural gas, respectively. This proves that the ASBR operation of DJW to produce hydrogen is an environmentally friendly process with the possibilities towards the greener and cleaner environment.

### 4.4. Significance of the Results: Outlook and Suggestions

The operation strategy for a lignocellulose based waste is an important step towards its commercialization of the technology, especially industrial waste like DJW. Increasing the total energy values (biodiesel, biohydrogen, and biomethane) from a single input low-cost waste, that is, jatropha, biomass would be a feasible solution for the future energy demands. Additionally, biodiesel production from jatropha biomass was demonstrated previously by various authors as a promising future energy carrier [34–36].

Comparison of the various lignocellulose biomass conversions to hydrogen using ASBR operation was given in Table 6. It could be seen that, in ASBR operation, HRT is mostly dependent on the substrate nature. For example DJW and marine algae are solid in nature have long HRT due to the long adaptation period of microbes to utilize the substrate. The production performance varied significantly among the substrates and the microbial source used. The HPR value of the DJW-ASBR operation is comparable with the other cellulosic wastes. However, the HPR and HY values of POME are higher than other values reported. This is mainly due to the high amount of nutrients present in it. Thus, hydrogen fermentation is directly proportional to the amount of sugars present in it. So, recovering the sugars in the form of hydrolysate is suggested to enhance the production performance of DJW.

In addition, in this study the operation strategies used have proved that ASBR operation was a good way to treat the DJW effectively and generate energy meanwhile. The utilization of the H_2_ fermentor effluent promised that more amount of bioenergy could be generated in the form of methane which is having higher heating value. This kind of approach to treat the solid waste is more suitable for industrial scale applications. On the whole bioenergy production from jatropha biomass and deoiled jatropha waste is the economically feasible and commercially applicable to solve the energy-related issues.

From the above results and discussions, direct conversion of De-oiled jatropha waste to hydrogen was demonstrated via ASBR operation and the effluent from H_2_ fermentor was efficiently utilized for methane production in batch tests using pig slurry as seed source and the following conclusions could be drawn:

 Stable hydrogen production/steady state was observed after 30 days of operation. The effluent from the reactor could be converted into methane gas to increase the total energy production of the process.
 Peak HPR and HY were attained as 1.48 ± 0.04 L/L∗d and 8.7 ± 0.3 mL H_2_/g volatile solid added when the reactor was operated at HRT 2 d with DJW concentration 200 g/L, temperature 55°C, and pH 6.5.
 Peak MPR and MY were achieved as 425.3 ± 5.1 mL/L∗d and a 20.5 ± 0.5 mL CH_4_/g COD, while Pig slurry was used as seed source with the effluent concentration of 10 g COD/L at 30°C and pH 7.0.
 This system demonstrated that ASBR operation could be a feasible method to treat the solid lignocellulose wastes such as DJW. PCR-DGGE results revealed the presence of combination of *Clostridium thermopalmarium* and *Bacillus coagulans* which are cellulolytic and fermentative in nature.

## Figures and Tables

**Table 1:** Operation strategies of the ASBR.

Run	Operation time	HRT (d)	Substrate loading rate (SLR)
1	1–48 hrs (2 days)	Batch (2 days)	Glucose (10 g/L)
2	2 days	Batch (2 days)	DJW (100 g/L)
3	5 days	2.5	DJW (200 g/L)
4	5 days	2.5	DJW (200 g/L)
5	10 days	2	DJW (200 g/L)
6	10 days	1.75	DJW (200 g/L)
7	10 days	1.5	DJW (200 g/L)

**Table 2:** Biogas production performance of the ASBR.

Run	BPR (L/L∗d)	H_2_%	HPR (L/L∗d)	HY (mL H_2_/g VS added)
1	4.32 ± 0.11	51.61 ± 0.70	2.2 ± 0.10	223.1 ± 8.9*
2	2.80 ± 0.12	44.63 ± 0.70	1.2 ± 0.10	14.6 ± 0.3
3	1.83 ± 0.26	29.96 ± 3.69	0.56 ± 0.13	3.3 ± 0.8
4	2.24 ± 0.09	35.40 ± 1.45	0.79 ± 0.03	4.7 ± 0.2
5	3.29 ± 0.08	44.93 ± 0.79	1.48 ± 0.04	8.7 ± 0.3
6	3.02 ± 0.09	44.97 ± 1.77	1.36 ± 0.02	7.9 ± 0.2
7	1.64 ± 0.24	30.56 ± 1.51	0.50 ± 0.07	2.9 ± 0.4

*Represents mL H_2_/g Glucose_added_.

**Table 3:** Effluent and SMP analysis at steady state.

Conditions HRT 2 days, SC: 200 g/L, pH: 6.5, T: 55°C			
Effluent analysis (g/L)			
TCOD	SCOD	TC	TS
14.4 ± 2.1	11.2 ± 1.3	9.3 ± 1.8	2.3.±0.8

SMP analysis (g/L)			
EtOH	HAc	HBu	HPr
0.68 ± 0.4	1.8 ± 0.2	2.3 ± 0.4	0.84 ± 0.6

TCOD: total chemical oxygen demand; SCOD: soluble COD; TC: total carbohydrate; TS: total solids; EtOH: ethanol; HAc: acetate; HBu: Buytrate; HPr: Propionate.

**Table 4:** Methane fermentation of H_2_ fermentor effluent.

Seed source	Final pH	Cumulative biogas	Cumulative CH_4_	MPR (mL/L∗d)	MY (mL CH_4_/g COD)
Pig slurry	6.8 ± 1.2	368 ± 4.4	201.0 ± 2.6	425.3 ± 5.1	20.5 ± 0.5
Cow dung	7.0 ± 1.0	259 ± 12.1	139.3 ± 5.7	348.6 ± 4.2	13.7 ± 0.8

**Table 5:** Affiliation of DGGE fragments determined by their 16S rDNA and isolated microorganisms.

Sequence no.	Family	Closest match	Homology (%)	Sequence length (bp)
1	Firmicutes	*Bacillus ginsengihumi* strain Gsoil 114	99	422
		(Accession no. NR_041378)		

2	Firmicutes	*Bacillus coagulans* strain NBRC 12583	98	419
		(Accession no. NR_041523)		

3	Firmicutes	*Clostridium thermopalmarium* strain BVP	99	411
		(Accession no. NR_026112)		

4	Firmicutes	*Clostridium thermopalmarium* strain BVP	97	411
		(Accession no. NR_026112)		

**Table 6:** Comparison with other cellulosic materials operated via ASBR operation.

Substrate	Seed source	HRT (h)^a^	Hydrogen production index	Reference
POME	Thermoanaerobacterium rich sludge	96	HPR: 6.1 L/L∗d, HY: 2.24 moL H_2_/moL hexose	[37]
Sweet sorghum extract	Indigenous microflora	12	HPR: 3.5 L/L∗d, HY: 0.93 moL/moL glucose	[38]
POME	Mixed microflora	72	HPR: 6.7 L/L∗d, HY: 0.94 L/g COD	[39]
Water hyacinth	Pig slurry	nr	HPR: 0.2 L/L∗d, HY: nr	[40]
DJW	Mixed microflora	36	HPR: 1.48 L/L∗d, HY: 8.6 mL/g VS	This study
Tequila vinasse	Anaerobic granular sludge	12	HPR: 2.12 L/L∗d, HY: nr	[10]
Food waste	Heat treated sludge	12	HPR: 7.6 L/L∗d, HY: 1.12 moL/moL hexose	[41]
Marine algae	Mixed microflora	144	HPR: nr, HY: 0.79 moL/moL hexose	[9]

nr: not reported in the source, ^a^calculated from the source.

## References

[1] Das D, Veziroğlu TN (2001). Hydrogen production by biological processes: a survey of literature. *International Journal of Hydrogen Energy*.

[2] Momirlan M, Veziroglu TN (2002). Current status of hydrogen energy. *Renewable and Sustainable Energy Reviews*.

[3] Pakarinen O, Lehtomäki A, Rintala J (2008). Batch dark fermentative hydrogen production from grass silage: the effect of inoculum, pH, temperature and VS ratio. *International Journal of Hydrogen Energy*.

[4] Zhang M-L, Fan Y-T, Xing Y, Pan C-M, Zhang G-S, Lay J-J (2007). Enhanced biohydrogen production from cornstalk wastes with acidification pretreatment by mixed anaerobic cultures. *Biomass and Bioenergy*.

[5] Levin DB, Carere CR, Cicek N, Sparling R (2009). Challenges for biohydrogen production via direct lignocellulose fermentation. *International Journal of Hydrogen Energy*.

[6] Lay C-H, Sung I-Y, Kumar G, Chu C-Y, Chen C-C, Lin C-Y (2012). Optimizing biohydrogen production from mushroom cultivation waste using anaerobic mixed cultures. *International Journal of Hydrogen Energy*.

[7] Sricharoenchaikul V, Atong D (2009). Thermal decomposition study on Jatropha curcas L. waste using TGA and fixed bed reactor. *Journal of Analytical and Applied Pyrolysis*.

[8] Srividhya KP, Tamizharasan T, Jayaraj S (2010). Characterization and gasification using-Jatropha Curcas Seed Cake. *Journal of Biofuels*.

[9] Shi X, Jung K-W, Kim D-H, Ahn Y-T, Shin H-S (2011). Direct fermentation of Laminaria japonica for biohydrogen production by anaerobic mixed cultures. *International Journal of Hydrogen Energy*.

[10] Buitrón G, Carvajal C (2010). Biohydrogen production from Tequila vinasses in an anaerobic sequencing batch reactor: effect of initial substrate concentration, temperature and hydraulic retention time. *Bioresource Technology*.

[11] Arooj MF, Han S-K, Kim S-H, Kim D-H, Shin H-S (2008). Effect of HRT on ASBR converting starch into biological hydrogen. *International Journal of Hydrogen Energy*.

[12] Kumar G, Lin CY (2013). Bio conversion of De-oiled Jatropha waste to hydrogen and methane gas by anaerobic fermentation: influence of substrate concentration, temperature and pH. *International Journal of Hydrogen Energy*.

[13] Endo G, Noike T, Matsumoto T (1982). Characteristics of cellulose and glucose decomposition in acidogenic phase of anaerobic digestion. *Proceedings of the Society For Civil Engineers*.

[14] (1995). *APHA Standard Methods for the Examination of Water and Wastewater*.

[15] Chen C-C, Lin C-Y, Lin M-C (2002). Acid-base enrichment enhances anaerobic hydrogen production process. *Applied Microbiology and Biotechnology*.

[16] Lin CY, Chang RC (1999). Hydrogen production during the anaerobic acidogenic conversion of glucose. *Journal of Chemical Technology and Biotechnology*.

[17] Koehler LH (1952). Differentiation of carbohydrates by anthrone reaction rate and color intensity. *Analytical Chemistry*.

[18] Owen WF, Stuckey DC, Healy JB (1979). Bioassay for monitoring biochemical methane potential and anaerobic toxicity. *Water Research*.

[19] Muyzer G, De Waal EC, Uitterlinden AG (1993). Profiling of complex microbial populations by denaturing gradient gel electrophoresis analysis of polymerase chain reaction-amplified genes coding for 16S rRNA. *Applied and Environmental Microbiology*.

[20] Tamura K, Dudley J, Nei M, Kumar S (2007). MEGA4: Molecular Evolutionary Genetics Analysis (MEGA) software version 4.0. *Molecular Biology and Evolution*.

[21] Saitou N, Nei M (1987). The neighbor-joining method: a new method for reconstructing phylogenetic trees. *Molecular biology and evolution*.

[22] Kumar G, Lay C-H, Chu C-Y, Wu J-H, Lee S-C, Lin C-Y (2012). Seed inocula for biohydrogen production from biodiesel solid residues. *International Journal of Hydrogen Energy*.

[23] Saraphirom P, Reungsang A (2011). Biological hydrogen production from sweet sorghum syrup by mixed cultures using an anaerobic sequencing batch reactor (ASBR). *International Journal of Hydrogen Energy*.

[24] Thauer RK, Jungermann K, Decker K (1977). Energy conservation in chemotrophic anaerobic bacteria. *Bacteriological Reviews*.

[25] Dabrock B, Bahl H, Gottschalk G (1992). Parameters affecting solvent production by *Clostridium pasteurianum*. *Applied and Environmental Microbiology*.

[26] Khanal SK, Chen W-H, Li L, Sung S (2004). Biological hydrogen production: effects of pH and intermediate products. *International Journal of Hydrogen Energy*.

[27] Chen C-Y, Yang M-H, Yeh K-L, Liu C-H, Chang J-S (2008). Biohydrogen production using sequential two-stage dark and photo fermentation processes. *International Journal of Hydrogen Energy*.

[28] Chuang Y-S, Lay C-H, Sen B (2011). Biohydrogen and biomethane from water hyacinth (*Eichhornia crassipes*) fermentation: effects of substrate concentration and incubation temperature. *International Journal of Hydrogen Energy*.

[29] Raheman H, Mondal S (2012). Biogas production potential of jatropha seed cake. *Biomass and Bioenergy*.

[30] Geng A, He Y, Qian C, Yan X, Zhou Z (2010). Effect of key factors on hydrogen production from cellulose in a co-culture of *Clostridium thermocellum* and *Clostridium thermopalmarium*. *Bioresource Technology*.

[31] Levin DB, Pitt L, Love M (2004). Biohydrogen production: prospects and limitations to practical application. *International Journal of Hydrogen Energy*.

[32] Walton SL, Bischoff KM, Van Heiningen ARP, Van Walsum GP (2010). Production of lactic acid from hemicellulose extracts by Bacillus coagulans MXL-9. *Journal of Industrial Microbiology and Biotechnology*.

[33] Sleat R, Mah RA, Robinson R (1984). Isolation and characterization of an anaerobic, cellulolytic bacterium, *Clostridium cellulovorans* sp. nov. *Applied and Environmental Microbiology*.

[34] Kumar Tiwari A, Kumar A, Raheman H (2007). Biodiesel production from jatropha oil (Jatropha curcas) with high free fatty acids: an optimized process. *Biomass and Bioenergy*.

[35] Berchmans HJ, Hirata S (2008). Biodiesel production from crude Jatropha curcas L. seed oil with a high content of free fatty acids. *Bioresource Technology*.

[36] Sahoo PK, Das LM (2009). Process optimization for biodiesel production from Jatropha, Karanja and Polanga oils. *Fuel*.

[37] O-Thong S, Prasertsan P, Intrasungkha N, Dhamwichukorn S, Birkeland N-K (2007). Improvement of biohydrogen production and treatment efficiency on palm oil mill effluent with nutrient supplementation at thermophilic condition using an anaerobic sequencing batch reactor. *Enzyme and Microbial Technology*.

[38] Antonopoulou G, Gavala HN, Skiadas IV, Lyberatos G (2010). Influence of pH on fermentative hydrogen production from sweet sorghum extract. *International Journal of Hydrogen Energy*.

[39] Badiei M, Jahim JM, Anuar N, Sheikh Abdullah SR (2011). Effect of hydraulic retention time on biohydrogen production from palm oil mill effluent in anaerobic sequencing batch reactor. *International Journal of Hydrogen Energy*.

[40] Lay CH (2012). *Bioenergy production potential of water hyacinth [Ph.D. dissertation]*.

[41] Kim S-H, Han S-K, Shin H-S (2006). Effect of substrate concentration on hydrogen production and 16S rDNA-based analysis of the microbial community in a continuous fermenter. *Process Biochemistry*.

